# Basal Forebrain Mediates Motivational Recruitment of Attention by Reward-Associated Cues

**DOI:** 10.3389/fnins.2018.00786

**Published:** 2018-10-30

**Authors:** Faezeh Tashakori-Sabzevar, Ryan D. Ward

**Affiliations:** Department of Psychology, University of Otago, Dunedin, New Zealand

**Keywords:** basal forebrain, sustained attention, reward-associated cues, inhibition, DREADD, cognitive effort

## Abstract

The basal forebrain, composed of distributed nuclei, including substantia innominata (SI), nucleus basalis and nucleus of the diagonal band of Broca plays a crucial neuromodulatory role in the brain. In particular, its projections to the prefrontal cortex have been shown to be important in a wide variety of brain processes and functions, including attention, learning and memory, arousal, and decision-making. In the present study, we asked whether the basal forebrain is involved in recruitment of cognitive effort in response to reward-related cues. This interaction between motivation and cognition is critically impacted in psychiatric conditions such as schizophrenia. Using the Designer Receptor Exclusively Activated by Designer Drug (DREADD) technique combined with our recently developed signaled probability sustained attention task (SPSA), which explicitly assays the interaction between motivation and attention, we sought to determine the role of the basal forebrain in this interaction. Rats were stereotaxically injected in the basal forebrain with either hM4D(Gi) (a virus that expresses receptors which silence neurons in the presence of the drug clozapine-N-oxide; CNO) or a control virus and tested in the SPSA. Behavior of rats during baseline and under saline indicated control by reward probability. In the presence of CNO, differential accuracy of hM4D(Gi) rats on high and low reward-probability trials was abolished. This result occurred despite spared ability of the reward-probability signals to differentially impact choice-response latencies and omissions. These results indicate that the basal forebrain is critical for the motivational recruitment of attention in response to reward-related cues and are consistent with a role for basal forebrain in encoding and transmitting motivational salience of reward-related cues and readying prefrontal circuits for further attentional processing.

## Introduction

Behavioral flexibility refers to the ability to adjust behavior adaptively in response to stimuli or changes in contingency in the environment. Deficits in behavioral flexibility contribute to functional impairment in psychiatric diseases such as schizophrenia (Heimer, 2000; Leeson et al., 2009). There are a number of regulatory circuits in the brain which contribute to behavioral flexibility. In particular, prefrontal cortical areas have been implicated (Duncan and Owen, 2000; Johnstone et al., 2007). These areas receive a direct projection from the basal forebrain (BF; including substantia innominata (SI), nucleus basalis, nucleus of the diagonal band of Broca and the nucleus basalis of Meynert (Woolf, 1991; Zaborszky, 2002; Zaborszky et al., 2012, 2015). This projection, comprising both cholinergic and GABAergic neurons (and a smaller glutamatergic projection), is one of the more important modulatory circuits in the brain (Gritti et al., 2006). The cholinergic projection has been shown to be involved in many cognitive and behavioral processes such as attention, learning and memory, arousal, decision-making, and cortical plasticity associated with cognitive performance (Mesulam et al., 1983; Bakin and Weinberger, 1996; Everitt and Robbins, 1997; Wenk, 1997; Kilgard and Merzenich, 1998; Risbrough et al., 2002; Weinberger, 2003; Sarter et al., 2005; Goard and Dan, 2009; Zaborszky et al., 2013; Anaclet et al., 2015; Ballinger et al., 2016). The GABAergic neurons, while relatively less studied than the cholinergic projections, have been shown to modulate cortical networks which are associated with cognition, and are thought to participate in decision-making processes (Sarter and Bruno, 2002; Lin et al., 2006; Zaborszky et al., 2012; Nguyen and Lin, 2014; Anaclet et al., 2015).

While many studies have focused on the neurobiology and contribution of BF and cortex in motivational and cognitive processes, there have been few studies of the role of the BF on explicit motivational modulation of cognitive effort. In particular, the contribution of BF and its connections in modulating the interaction between motivation and attention remains poorly understood. Understanding this interaction is of particular importance given the significant impact of attention on other cognitive processes, and also data indicating that cognition and motivation likely interact to produce dysfunctions in psychiatric patients (Barch, 2005; Barch and Dowd, 2010).

**FIGURE 1:**
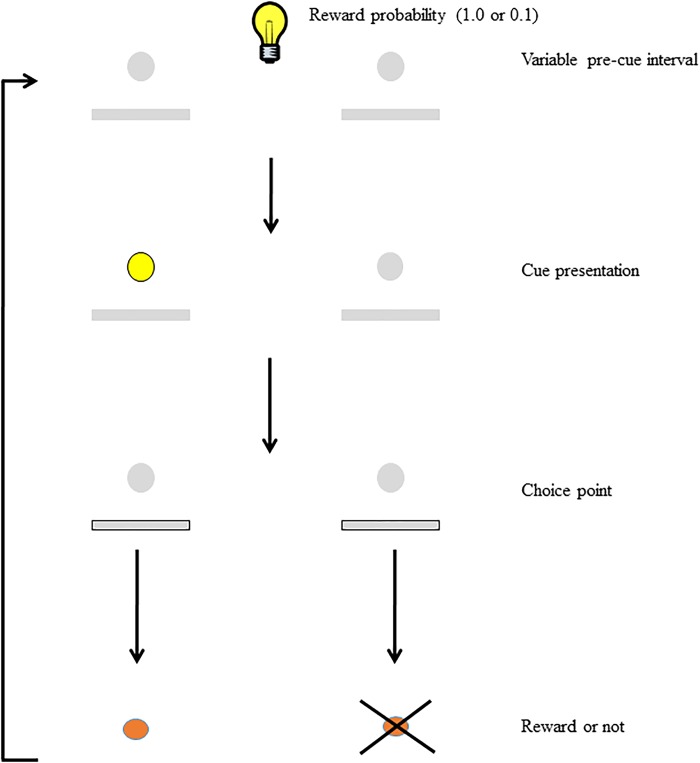
Schematic of the SPSA task (after Hall-McMaster et al., 2017). The houselight state during the variable pre-cue interval (either on or off) signals whether the probability of reward for a correct response is high (1.0) or low (0.1). Following the pre-cue interval, the cue light signals which lever will be rewarded at the choice point. Both levers are extended and correct responses are rewarded (or not) according to the probability signaled at the beginning of the trial.

To study the interaction between motivation and attention, we developed the Signaled Probability Sustained Attention (SPSA; Figure 1) task (Ward et al., 2015a,b; Hall-McMaster et al., 2017; Bates et al., 2018). In this prefrontal-dependent sustained-attention task (Kahn et al., 2012) rats must attend to cues that signal which of two responses will be rewarded on each trial. We manipulate motivation in this task on a trial-by-trial basis by signaling to the rat the probability of reward for correct responses. In our previous studies we have shown that discrimination accuracy on high reward-probability trials is higher than accuracy on low reward-probability trials, and we have interpreted this as evidence that the reward probability signal acts as a cognitive incentive, leading to increased cognitive effort on high reward-probability trials. In addition, rats are more motivated to respond on high reward-probability trials than on low, as indicated by differential impacts on choice-response latency and omissions. Thus, the SPSA provides a way to interrogate and differentiate the impact of manipulated variables on motivation, attention, and their interaction.

We have previously shown that prefrontal cortex (specifically orbitofrontal cortex; OFC) and striatum are critical for the ability of reward probability signals to modulate attention in our task (Ward et al., 2015a,b; Hall-McMaster et al., 2017). Dysfunction of these areas (either induced virally or via a transgenic method) abolished the modulatory ability of reward probability signals on accuracy. Furthermore, this effect took place in the absence of overall decreases in accuracy, suggesting that the impact was specifically on the motivational modulation of behavior, rather than a more fundamental effect on attentional processes. Finally, the reward-probability signals still exerted some motivational influence, as choice-response latencies and omissions reflected the signaled reward probability. These results indicate that deficient OFC and striatal function in this task affects the translation of motivational information conveyed by the reward-probability signals into adaptive choice responding, rather than having some general effect on attention or motivation *per se*.

The BF is situated at a critical juncture in a network that subserves both motivation and attention. The largest output projection from the ventral striatal area most critical to reward-motivated behavior, the nucleus accumbens, is a GABAergic projection to the BF. In turn, the BF sends direct projections (both GABAergic and cholinergic) to the prefrontal cortex (Gritti et al., 2006). The cholinergic projection has been shown to be critical to accurate cue detection and attention performance (Parikh et al., 2007; Peters et al., 2011; Gritton et al., 2016). As an intermediary between the nucleus accumbens and the prefrontal cortex, the BF is situated well to play a role in the motivational modulation of attention in our task. In addition, a subset of the non-cholinergic (putatively GABAergic) BF projections have been shown to encode motivational salience of reward-related stimuli and transiently support cortical information processing related to online decision making (Lin and Nicolelis, 2008), thus providing a putative mechanism for the dynamic modulation of attention seen in our task. Here, we trained rats in the SPSA task and inhibited BF neural activity during task performance by means of the Designer Receptors Exclusively Activated by Designer Drugs (DREADD) method. Our aim was to determine the role of the BF in motivational modulation of attention performance by signaled reward probability.

## Materials and Methods

All experimental procedures reported here were approved by the University of Otago Animal Ethics Committee.

### Subjects

Twenty Four adult male Long-Evans rats (12 weeks old) obtained from the Hercus Taieri Resource Unit. All the subjects (three rats per cage) were housed under a 12-h, light-dark (6 am to 6 pm) cycle in a temperature -controlled room (22 C ± 1 C).

### Apparatus

10 identical operant chambers (controlled through Med-associates, St. Albans, VT: model ENV-008w) with internal dimensions of 30.5 cm length × 24.1 cm width × 21.0 cm height were used in this experiment. The hinged door, ceiling and rear panel of the apparatus were constructed from clear polycarbonate while both end walls, grid floor (0.87 cm rods spaces) and waste pan were constructed of stainless steel. Each chamber was equipped with cue lights above each of two retractable levers, a house light (for chamber illumination), a speaker (for presenting signal tones before delivery of reward) and a food hopper (with infrared photocell detector). Each operant box was housed in a light and sound-attenuating chamber that contained a fan that provided 72 dB masking noise. Experimental events were controlled and data recorded using a dedicated computer running MedPC IV programs and software.

### Procedure

#### Surgery

Viruses were obtained from the University of North Carolina Gene Therapy Center Vector Core. BF coordinates were adapted from Paxinos and Watson (2007) and in consultation with previous studies (Quinn et al., 2010; Baxter et al., 2013; Avila and Lin, 2014b; Nair et al., 2016). Each rat was anesthetized with isoflurane and then stereotaxically injected bilaterally with 1 μl virus into the target region (AP: 0.6 mm, ML: 2.25, DV: 7.6 from the dura). AAV2/hSyn-HA-hM4D(Gi)-IRES mCitrine; 5.6 × 10^12^ particles/ml; hereafter referred to as hM4D(Gi) and AAV2/hsyn-eGFP (33 x 10^12^ particles/ml; hereafter referred to as GFP) viruses were injected by a custom-made needle connected to a Hamilton syringe at an infusion rate of 0.25 μl/min.

Following surgery, animals were housed individually for 5 days. Then subjects were returned to the normal cages (three rats in each cage) and given 1-week recovery time before initial food deprivation. Behavioral training commenced after rats’ weights reached 85–90% base weight and they were kept under food deprivation during the experiment.

### Behavioral Procedures

On two consecutive days before training began, rats were given a capful of the food pellets to be used as a reward in the experiment in their home cages to accustom them to eating the pellets. All experimental sessions took place at the same time of day, during the light phase, one session per day and 7 days per week. The sequence of behavioral procedures followed our previously published protocol (Hall-McMaster et al., 2017).

#### Lever-Press Training

Rats were first trained to consume reward pellets from the food hopper in two sessions in which 60 rewards were delivered on a variable interval schedule (mean interfood interval = 30 s, range = 0.76–119.87 s). Rats were then trained to press levers for reward under a continuous reinforcement schedule. During these sessions, either the left or right lever was extended for 10 s and a lever press resulted in reward delivery and the initiation of an intertrial interval (mean duration = 30 s, range = 0.76–119.87 s). Sessions were comprised of 30 left and 30 right lever presentations (presented pseudorandomly) and continued for 60 trials. Rats received three sessions, after which they all responded on more than 50 out of 60 trials.

#### Single Cue-Single Lever Training

During single cue-single lever training, rats received trials where a cue light above either the left or right lever was illuminated for 10 s. One second after the cue terminated, the lever in the cued position was extended and a lever press was rewarded. If no response was made after 10 s, the trial ended and a new trial began. Between trials, there was a variable interval (mean = 45 s; range = 2.74–148.13 s) which required rats to sustain attention to the stimulus arrays to detect illumination of the cue light. Sessions lasted for 68 trials. Rats received three sessions of single-cue-single lever training, one each of only left and right cue and lever presentations, and one which consisted of 50% left and 50% right cue and lever presentations. On the third day of training, all rats pressed the cued lever on at least 65 out of 68 trials.

#### Choice Training

During choice training, a percentage of trials were single-cue single-lever trials as described above, and the remainder were choice trials, in which either the left or right lever was cued for 5 s and then both levers were presented. A response on either lever within 10 s resulted in retraction of both levers and a response to the previously cued lever was rewarded. Choice and single-cue single-lever trials were randomly intermixed. Rats received three sessions each of 50, 75, and 100% choice trials. During this training phase, incorrect responses initiated a correction procedure, in which the trial was repeated with the same cue location until it was completed correctly. Next, rats received three sessions of 100% choice trials with no correction, following which all rats were responding correctly on more than 65 out of 68 trials. The cue duration was then reduced from 5s to 1s over the course of 7 sessions.

#### SPSA Training

During this phase, rats performed the sustained attention task as described above (see Figure 1), but the probability of reward for a correct response (1.0 or 0.1) was signaled by turning the houselight either on or off (counterbalanced across rats) for the duration of the trial until a choice response occurred. Rats received an equal number of high and low probability trials presented with the constraint that no more than four trials of the same type could be presented in a row. Rats were trained on this version of the task for 25 sessions.

Following acquisition of SPSA performance, rats received one i.p. injection of saline to accustom them to the injection procedure. Following this, rats received injections of saline and CNO (2 mg/kg; volume 0.5 ml/kg) 30 min before the daily session. Order of injections was counterbalanced and injections were given according to a within-subjects design, in which all rats received both saline and CNO. Previous *in vitro* and *in vivo* DREADD studies, including our own work, have shown that CNO in the absence of hM4D(Gi) has no effect on neuronal activity, while in presence of hM4D(Gi) receptors, CNO causes hyperpolarization of neurons. In addition, DREADDs have been shown to modulate BF neuronal activity in mice and rats (Armbruster et al., 2007; Nichols and Roth, 2009; Gremel and Costa, 2013; Chang et al., 2015; Nair et al., 2015; Hall-McMaster et al., 2017).

### Histology

Following the conclusion of the experiment, rats were deeply anesthetized and perfused with paraformaldehyde and brains processed for histology to verify injection location and spread of viral infection. Viral expression was visualized using a fluorescent microscope.

### Data Analysis

The main dependent variable of interest was the proportion of correct responses on high and low reward-probability trials. Latency to make a choice response, latency to retrieve reward, the number of omissions on high and low reward-probability trials, and the number of perseverative responses and errors was also measured. Data were analyzed by repeated measures ANOVA with *post hoc* comparisons carried out using Bonferonni corrections.

## Results

### Equal Performance of GFP and hM4D(Gi) Rats Before Manipulation

**FIGURE 2:**
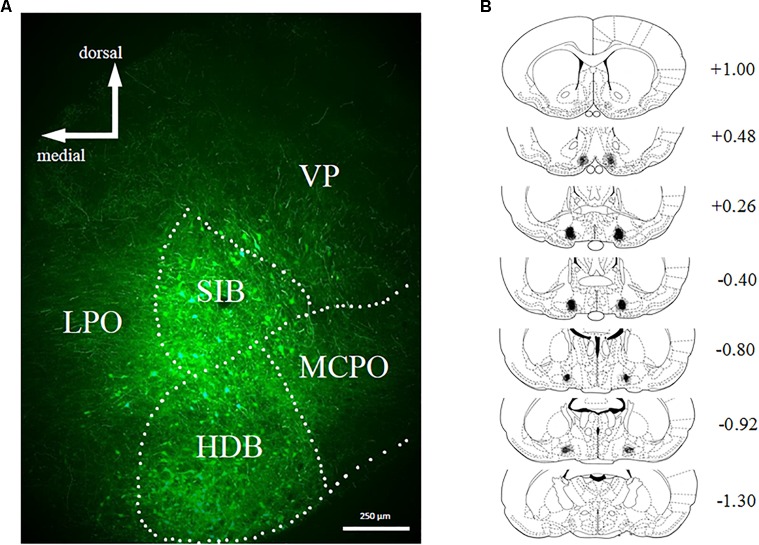
**(A)** Representative example of viral expression in basal forebrain. **(B)** Graphic representation of viral spread [minimal (black) and maximal (gray)] in basal forebrain. Numbers represent the anteroposterior distance from bregma according to Copyright© 2007 George Paxinos and Charles Watson by Elsevier Academic Press. Adapted with permission. hM4D(Gi) *N* = 12, GFP *N* = 12.

Bilateral stereotaxic injection of hM4D(Gi) and GFP mCitrine expressing adeno-associated viruses resulted in neuron-specific expression in BF due to the use of the human synapsin promotor (hSyn). Figure 2A shows a representative image of viral expression in the BF. Figure 2B shows the minimal (black) and maximal (gray) extent of intrinsic fluorescence of mCitrine (a marker of the hM4D(Gi)-expressing virus). Viral expression was focused on substantia innominata and the horizontal limb of the diagonal band, with some expression occurring in magnocellular preoptic nucleus and lateral preoptic area. There were no systematic differences in the viral expression pattern in hM4D(Gi) or GFP groups.

**FIGURE 3:**
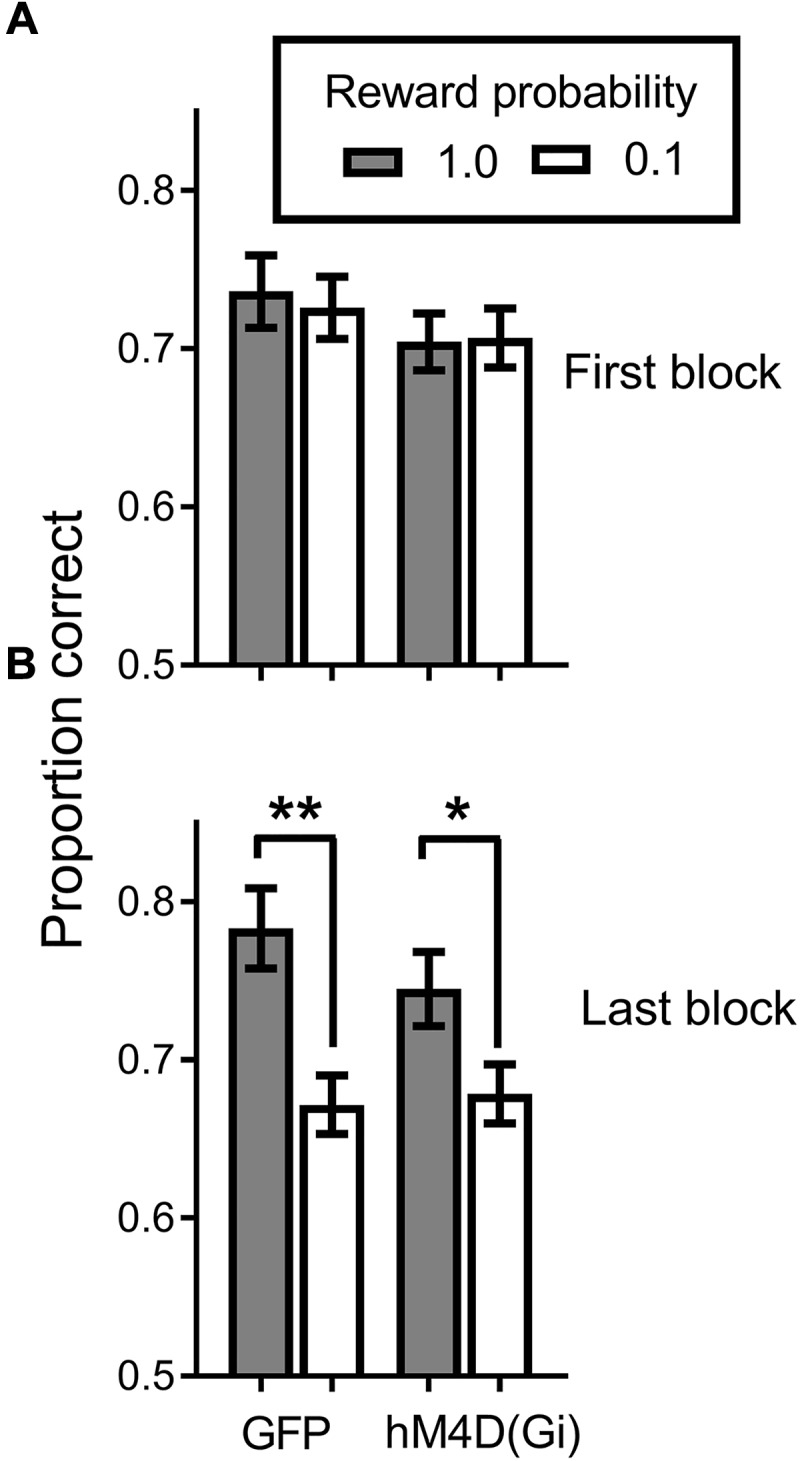
Proportion correct during the signaled probability sustained attention (SPSA) task for hM4D(Gi) and GFP rats during acquisition. **(A)** Proportion correct during the first three session block of exposure to the SPSA task. **(B)** Differentiation of accuracy on high and low reward-probability in the last three session block of SPSA task acquisition. hM4D(Gi) *N* = 12, GFP *N* = 12. ^∗∗^*p* < 0.01, ^∗^*p* < 0.05.

All rats acquired the discrimination task. Figure 3A shows that at the end of initial training, when the reward probability signals were introduced, discrimination accuracy was comparable (around .7 proportion correct) for hM4D(Gi) and GFP groups, with no difference in accuracy on high and low reward-probability trials. By the end of training with differential reward-probability signals (Figure 3B), there was a clear differentiation of accuracy on high and low reward-probability trials. These impressions of the figure were confirmed by a repeated measures ANOVA with virus (hM4D(Gi)/GFP) as a between-subjects factor and training block (initial/last) and reward probability (high/low) as within-subjects factors. The ANOVA found a significant effect of training block[F (1,22) = 11.587; *p* = 0.003], but not of reward probability [F (1,22) = 0.020; *p* = 0.888]. There was however, a significant training block x reward probability interaction[F (1,22) = 20.707; *p* = 0.000], as indicated by the differentiation between performance on high and low reward-probability trials during the last block of SPSA training. No other main effects or interactions were significant (F < 1.5). *Post hoc* comparisons showed a significant difference between accuracy on high and low reward probability trials during the last block for both GFP [t (11) = 3.818; *p* = 0.003] and hM4D(Gi) [t(11) = 2.718; *p* = 0.02] rats.

### Inhibition of BF Neuronal Activity Abolishes the Impact of Signaled Reward Probability on Discrimination Accuracy

**FIGURE 4:**
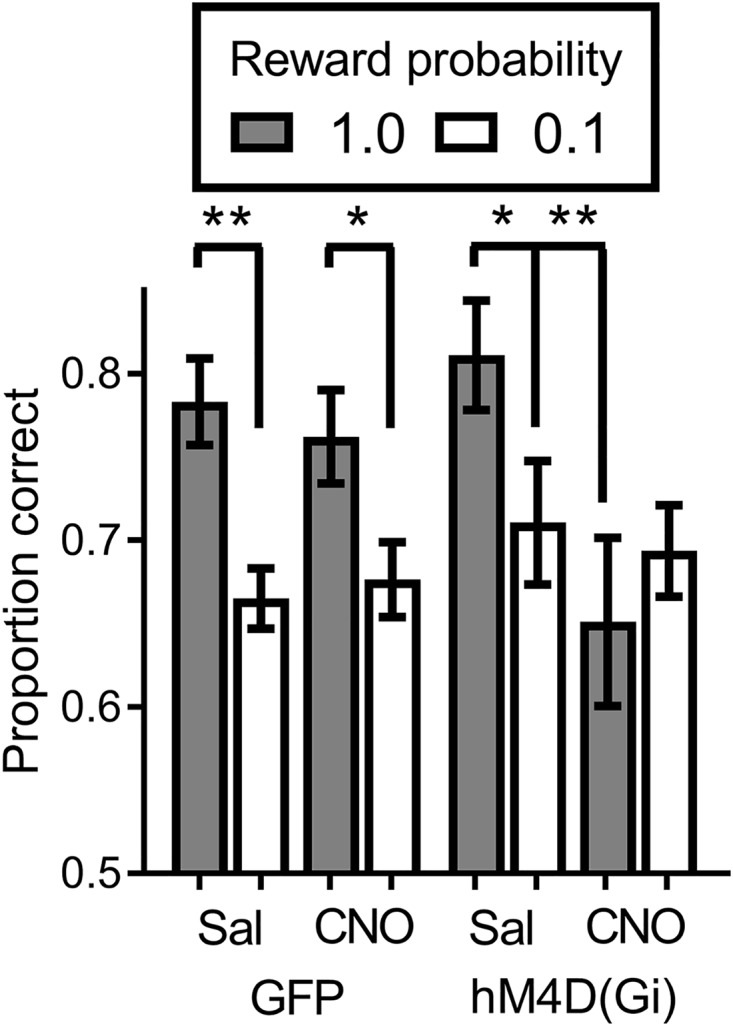
Proportion correct during the SPSA task for hM4D(Gi) and GFP rats after treatment with saline and CNO. hM4D(Gi) *N* = 12, GFP *N* = 12. ^∗∗^*p* < 0.01, ^∗^*p* < 0.05.

We tested virally injected rats in the SPSA task after injection of either saline or CNO. A reward probability × viral injection × treatment ANOVA on proportion correct (Figure 4) indicated that the effect of viral injection [GFP/hM4D(Gi)] [F (1,22) = 0.029; *p* = 0.865] and treatment [saline/CNO] [F (1,22) = 3.681; *p* = 0.068] were not significant. There was a significant effect of reward probability [F (1,22) = 15.149; *p* = 0.001] and a significant reward probability × treatment interaction [F (1,22) = 8.541; *p* = 0.008]. To test the nature of the significant interaction, we conducted separate reward probability × treatment ANOVAs on the data from the GFP and hM4D(Gi) rats. For GFP rats, the ANOVA found only a significant effect of reward probability [F (1,11) = 30.578; *p* = 0.000]; all other F < 1.0. Planned comparisons showed that there was a significant difference in accuracy following administration of saline [t (11) = 4.55; *p* = 0.0008] and CNO [t (11) = 4.34; *p* = 0.0012]. For the hM4D(Gi) rats, the ANOVA found only a significant reward probability x treatment interaction [F (1,11) = 7.20; *p* = 0.021]. Planned *post hoc* comparisons found that accuracy on high and low-reward probability trials was significantly different following administration of saline [t(11) = 2.494; *p* = 0.029] but not CNO [t(11) = 1.149; *p* = 0.275]. Furthermore, BF inhibition acted by selectively decreasing accuracy on high reward-probability trials, indicated by a significant difference between high reward-probability [t (11) = 3.109; *p* = 0.009] but not low reward-probability [t (11) = 0.355; *p* = 0.729] trials in sessions preceded by saline and CNO injections.

### Current Trial Reward Probability Selectively Dictates Discrimination Accuracy

**FIGURE 5:**
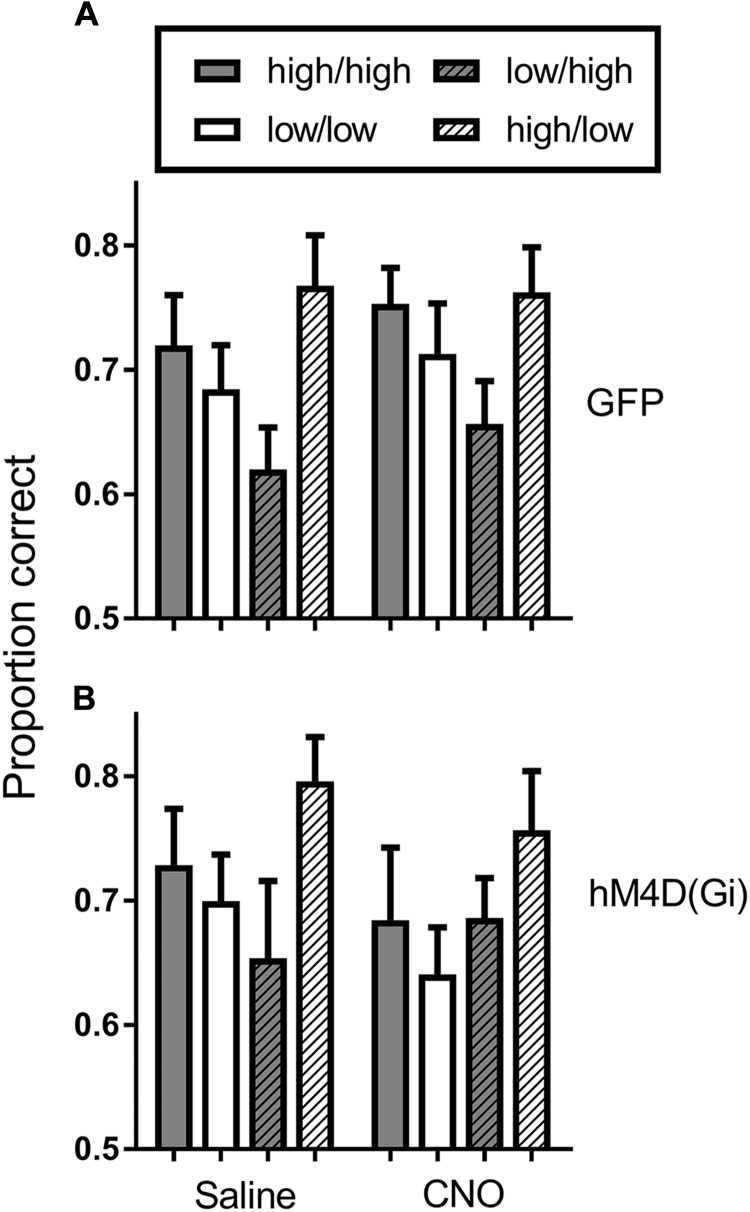
Average proportion correct on the current trial as a function of reward probability on the previous trial for hM4D(Gi) and GFP rats treated with saline and CNO. The first designation in each legend pair indicates the reward probability on the current trial, and the second indicates the reward probability on the previous trial. Thus, high/high indicates a high reward probability trial preceded by a high reward probability trial. hM4D(Gi) *N* = 12, GFP *N* = 12.

To further assess potential cognitive mechanisms of BF inhibition on motivational modulation of discrimination accuracy, we conducted a trial-by-trial analysis of the data during sessions preceded by saline and CNO administration. This analysis was conducted to determine whether performance on the current trial was governed by the reward probability on the current trial, or whether there was some bleedover in the influence of the reward probability on the previous trial. Figure 5 shows the results of the analysis. In general, discrimination accuracy was greatest on high probability trials and lower on low probability trials regardless of the prior trial reward probability. The exception to this general pattern was the hM4D(Gi) group treated with CNO. A 2 (current trial reward probability) × 2 (previous trial reward probability) x 2 (saline vs. CNO) repeated measures ANOVA with viral injection as a between groups factor found only a significant effect of current trial reward probability [F (1, 22) = 10.095; *p* = 0.004]; all other *p* = > 0.06. Separate ANOVAs were also conducted on the data from the GFP and hM4D(Gi) rats. These analyses found a significant effect of current trial reward probability for GFP [F (1,11) = 16.415; *p* = 0.002] but not hM4D(Gi) [F(1,11) = 2.648; *p* = 0.132] rats (all other main effects and interactions non-significant; *p* > 0.12). These data, together with the data discussed above further underscore that discrimination accuracy under BF inhibition was not differentially impacted by the reward-probability signals.

### Inhibition of BF Leaves Motivational Significance of Reward-Probability Signals Intact and Does Not Impact Perseverative Responding

We recorded and analyzed the number of omissions, the latency to make a choice response, and the number of perseverative responses and errors. These data are shown in Table 1. The omission data show that, in general, rats made very few omissions, however, they omitted significantly more low than high reward-probability trials [F(1,22) = 7.406; *p* = 0.012]. There were no significant differences between groups with different viral treatments [F (1,22) = 0.718; *p* = 0.406] and no differences between rats treated with saline or CNO [F (1,22) = 1.736; *p* = 0.201]. A similar result was obtained with respect to the choice-response latency data. In all cases rats were slower to respond on low than on high reward-probability trials [F(1,22) = 11.881; *p* = 0.002] and there was no difference in performance between GFP and hM4D(Gi) rats [F(1,22) = 0.020; *p* = 0.889] or rats treated with saline or CNO [F (1,22) = 0.672; *p* = 0.421].

**Table 1:** Average number of omissions, choice-response latency (sec), proportion perseverative responses and errors, and reward retrieval latency (standard error in parentheses).

Groups/Injections	Omission		Choice latency		Perseveration		Reward latency
	High	Low	High	Low	Responses	Errors	
GFP-Saline	0.41 (0.22)	1.16 (0.38)	0.52 (0.03)	0.70 (0.06)	0.25 (0.01)	0.09 (0.01)	1.18 (0.14)
GFP-CNO	0.50 (0.33)	0.66 (0.41)	0.59 (0.05)	0.80 (0.11)	0.25 (0.01)	0.07 (0.01)	1.17 (0.10)
hM4D-Saline	0.33 (0.33)	1.00 (0.66)	0.60 (0.07)	0.70 (0.08)	0.25 (0.01)	0.08 (0.01)	1.07 (0.08)
hM4D-CNO	0.00 (0)	0.16 (0.16)	0.61 (0.06)	0.65 (0.06)	0.23 (0.01)	0.08 (0.01)	1.15 (0.11)


To assess whether BF inhibition impacted perseverative responding, we calculated the number of responses to a previously rewarded lever, as well as the number of error responses to a previously rewarded lever. Around one quarter of responses were made to a previously rewarded lever. This proportion did not differ as a function of viral treatment [F(1,22) = 1.188; *p* = 0.288] or injection [F(1,22) = 1.010; *p* = 0.326]. Similarly, the proportion of perseverative errors was low and did not differ significantly across GFP or hM4D(Gi) rats [F(1,22) = 0.004; *p* = 0.950] or as a function of saline or CNO treatment [F(1,22) = 1.096; *p* = 0.307]. Finally, with respect to latency to retrieve reward, there was no impact of either viral injection [F(1,22) = 0.205; *p* = 0.655] or treatment [F(1,22) = 0.209; *p* = 0.652]. Together, these results indicate that although inhibition of BF neural activity abolished the ability of reward-probability signals to differentially impact discrimination accuracy, the motivational significance of these signals was left intact, as behavior on high and low reward-probability trials still reflected an appreciation of the motivational valence of the signals.

## Discussion

Inhibition of BF neuronal activity abolished the ability of reward-related cues to differentially modulate discrimination accuracy. Critically, the effects were only seen in the presence of CNO and the hM4D(Gi) receptors. Neither CNO or the presence of the hM4D(Gi) receptors alone had any impact on behavior. Further analyses showed that BF inhibition did not affect overall attention, as only accuracy on high reward-probability trials was impacted. BF inhibition also did not affect perseverative responding, and the choice-response latency and omission data indicated that the different reward-probability signals still produced behavioral effects, indicating that the effect of BF inhibition on discrimination accuracy was not due to an inability to appreciate the significance of the different reward probability signals. Rather, the effects were due to an inability to use information provided about reward probability to specifically modulate discrimination accuracy.

The present results extend those from other investigations of the role of the BF in attention-based decision making. While traditional ideas about the role of the BF focused on its diffuse projections and posited an overall modulatory role that takes place on a relatively longer time scale (Pribram and McGuinness, 1975; Briand et al., 2007; Sarter et al., 2009b), subsequent studies indicated that, in particular, the cholinergic projections to the prefrontal cortex played a critical role in specific and dynamic aspects of attention performance (Sarter et al., 2009a). This work demonstrated unequivocally and elegantly that the BF cholinergic projection mediates cue detection (defined as a behaviorally appropriate response to the presentation of a cue; a “hit” in the parlance of signal detection theory). In a sustained attention task similar to the one we used here (without differential reward probabilities and signals), Sarter et al. (2013) have shown that on trials in which a cue is detected, a cholinergic transient is evoked. On trials in which a cue is missed, the transients do not occur. They further showed that these cholinergic transients are causal in cue detection (Gritton et al., 2016). Using optogenetic methods, they stimulated BF cholinergic transmission and found that not only did accuracy of cue detection increase with increased cholinergic transmission, but stimulating BF cholinergic neurons on trials in which a cue was not presented led to the mice reporting that a cue had been seen, a “false alarm.” Thus, cholinergic transmission to prefrontal cortex is critical to the detection of cues in attention paradigms.

In the present study, the fact that accuracy was impacted only on high reward-probability trials, with no overall decrease in accuracy, indicates that the effects of BF inhibition in the present experiment did not impact cue detection, at least not at a global level. There are several possible reasons for this result. One potential reason is that the hM4D(Gi) virus used in the present experiment, while neuron specific, is not selective to specific neuron subtypes. Therefore, inhibition in our study possibly impacted all BF neuron types and we are not able to conclusively determine whether our results are due to the inhibition (or lack thereof) of a specific subpopulation of BF neurons or to a combination of factors which result from a more global inhibition.

While they have been much more studied than other populations of BF neurons, cholinergic neurons make up only a small fraction (around 6%) of the projection neurons in the BF, with the vast majority of identified neurons being GABAergic(Gritti et al., 2006). In contrast to results indicating the specific function of cortical cholinergic signaling in cue detection, BF manipulations that have preferentially impacted GABAergic neurons result in quite different and characteristic behavioral deficits. For example prior work has shown that lesions of BF with ibotinic acid preferentially targeted GABAergic neurons (Everitt et al., 1987; Robbins et al., 1989; Dunnett et al., 1991; Sarter and Dudchenko, 1991; Bednar et al., 1998) and led to a very specific behavioral impairment in a sustained-attention task similar to the one we employed here (Burk and Sarter, 2001). The task was composed of signal and non-signal trials and rats were rewarded for both “hits” and “correct rejections.” Lesions of BF with ibotinic acid did not affect the proportion of “hits” but led to an increase in the proportion of “false alarms.” Further analysis of the data indicated that this increase in false alarms was likely due to an inability of the rats to switch flexibly between rules for signal and non-signal trials. Thus, the authors concluded that GABAergic neurons were involved in more executive function type cognitive aspects of the attention performance, while cholinergic neurons were involved only in behavioral detection of cues (see also Sarter and Bruno, 2002). Given the lack of an impact on overall discrimination accuracy seen here, our results suggest that BF inhibition impacted some other aspect of cognitive performance aside from cue detection, and thus, most likely preferentially impacted GABAergic BF neurons.

The selective impact of BF inhibition on accuracy during high reward-probability trials may be explained by results which indicate that a putative population of BF GABAergic neurons are critically involved in signaling motivational salience of cues that are associated with behaviorally relevant outcomes (Lin et al., 2006; Lin and Nicolelis, 2008; Avila and Lin, 2014a,b; Raver and Lin, 2015). Lin and Nicolelis (2008) recorded from putative non-cholinergic BF neurons during a Go/No go task in which rats either made or withheld a licking response in response to presentation of either a tone or a light which had been associated with a specific outcome (sucrose or quinine). Upon presentation of the cue, BF neurons showed a rapid and transient bursting response, which the authors showed encoded the motivational salience of the reward-related cues. These cues only produced the burst responses following learned association with the outcomes. Furthermore, in a tone-discrimination task, these same neurons only showed burst-responding on trials in which tones were detected, as defined above by a cue-evoked appropriate behavioral response. The authors suggested that because BF GABAergic neurons preferentially synapse on cortical GABAergic interneurons, the bursting response by GABAergic neurons to motivationally salient cues could lead to rapid disinhibition of cortical circuits, essentially readying them for other attentional or cognitive processing requirements (Raver and Lin, 2015).

The different time scales of the cholinergic transients which control cue detection (on the order of seconds) and the putative GABAergic burst responses that signal motivational salience (on the order of milliseconds) suggests the intriguing possibility that cues that are endowed with motivational salience (through prior learning) lead to rapid cortical disinhibition upon presentation, which frees up prefrontal circuitry to further process attention-related cue information transmitted by cholinergic neurons, leading to a behaviorally appropriate response (a cue detection as defined above). If DREADD inhibition preferentially targeted non-cholinergic neurons in the present study, one interpretation of the present results is that BF inhibition impacted the ability of GABAergic neurons to burst in response to high reward-probability signals. These signals may have been differentially impacted due to the increased salience they had been endowed with through the process of training. Thus, without the bursting response to signal the motivational salience of high reward-probability signals and ready prefrontal circuitry to process the upcoming stimulus cues, these cues were not behaviorally detected, leading to a selective decrease in accuracy on high reward-probability trials. This interpretation of specific effects of BF inhibition on downstream cortical cognitive networks would also explain why the reward probability signals were still able to produce different behavioral effects on latencies and omissions, but not on discrimination accuracy.

While appealing, this interpretation is almost certainly incomplete. The complex connections of both cholinergic and non-cholinergic neurons with prefrontal areas lead to interactions between excitatory and inhibitory transmission that are not well understood (Sarter and Bruno, 2002). Furthermore, recent work has demonstrated that BF connections with other areas may be involved in the influence of reward-related cues on cognitive performance. For example a recent study implicated the pathway from the amygdala to the BF as a key player in aspects of spatial attention that are influenced by reward (Peck and Salzman, 2014) and earlier work implicated the amygdala in sustained-attention performance (Holland, 2007). In addition, aside from sending direct projections to prefrontal areas, the BF also receives projections from these areas (Carnes et al., 1990; Gaykema et al., 1991a,b; Ghashghaei and Barbas, 2001). Of particular interest regarding the present data are the reciprocal connections between OFC and BF (Ghashghaei and Barbas, 2001). It is well known that the OFC plays a critical role in value-based decision making (Schoenbaum et al., 2009; Schoenbaum et al., 2011; Takahashi et al., 2013). We recently found that disrupting OFC function abolished control of discrimination accuracy by signaled reward probability in our task (Ward et al., 2015b; Hall-McMaster et al., 2017) similarly to what we found here. Finally, other signaling pathways in the BF-prefrontal circuit, such as the endocannabinoid system, have recently been identified as important in BF modulation of cortical activity and in the processing of reward-driven motivational salience (Llorente-Ovejero et al., 2017; Brancato et al., 2018). Clearly, further work that delineates the specific neural populations and timecourse of neural activity involved in SPSA performance is needed in order to clarify the specific role of cholinergic and non-cholinergic BF projections neurons and other circuitry in the effects seen here.

Regardless of the specific mechanisms involved here, our results indicate that BF is necessary for the ability of reward-probability signals to modulate discrimination accuracy. These results further indicate that BF is not involved in the ability to process motivationally salient signals in our paradigm *per se*, but rather is required to translate motivational salience of reward probability signals into increased attentional performance. Together, these results shed further light on the role of the BF in behavioral flexibility. They also provide further evidence that, contrary to its traditionally held view as a diffuse and slow modulator of different arousal states, BF activity is involved in dynamic modulation of cognitive processing in response to behaviorally relevant and motivationally salient environmental cues.

## Author Contributions

RW conceptualized the research, provided laboratory resources, and material support for the research. FT-S conducted the research under supervision of RW. All authors analyzed the data and wrote the manuscript.

## Conflict of Interest Statement

The authors declare that the research was conducted in the absence of any commercial or financial relationships that could be construed as a potential conflict of interest.
